# Comparison of percutaneous endoscopic and standard open mini-hemilaminectomy in cats – a prospective, controlled, paired cadaver study

**DOI:** 10.1186/s12917-025-05147-3

**Published:** 2025-11-15

**Authors:** Alina Busch, Nina Dorothee Lorenz, Yury Zablotski, Susanne Lauer, Matthias Kornmayer

**Affiliations:** 1 Centre for Clinical Veterinary Medicine, LMU Small Animal Clinic, Ludwig- Maximilians-Universität München, Munich, Germany; 2 Northern Rivers Veterinary Specialists, Bangalow, Australia

**Keywords:** Endoscopy, Minimally invasive spine surgery, Mini-hemilaminectomy, Intervertebral disc disease, Feline

## Abstract

**Background:**

The purpose of this study was to evaluate the feasibility and operative outcomes of percutaneous endoscopic mini-hemilaminectomy (PE-MHL) compared to standard open mini-hemilaminectomy (SO-MHL) in feline cadavers. A prospective, controlled, paired cadaver study was conducted using fifteen skeletally mature domestic short-haired cat cadavers (median body weight 3.9 kg). At the L4–L5 intervertebral space, PE-MHL was performed on the left and SO-MHL on the right side. Times for fluoroscopic localization, approach, spinal cord exposure and closure were recorded. Incision length, suture length, and bone window area were measured. Bone windows were assessed by postoperative CT. Mixed-effects models compared techniques and evaluated procedure times across cases as a potential indicator of surgical improvement.

**Results:**

All 30 procedures provided full visualization of spinal cord, nerve root and ventral aspect of the vertebral canal. Mean ± standard error of the mean (SEM) of total surgical time was longer for PE-MHL than SO-MHL (65.4 ± 3.5 min vs. 30.3 ± 0.2 min), owing chiefly to longer exposure time; modelling predicted time parity after 20 PE-MHL cases. PE-MHL produced smaller skin incisions (8.5 ± 0.2 mm vs. 22.1 ± 0.5 mm), closure lengths (11.4 ± 0.5 mm vs. 23.1 ± 0.7 mm) and bone-window areas (51.3 ± 1.7 mm² vs. 64.7 ± 2.2 mm²). PE-MHL resulted in a single nerve-root injury. No other complications were observed.

**Conclusions:**

Percutaneous endoscopic mini-hemilaminectomy is technically feasible in the feline lumbar spine. Procedure times decreased with increasing number of cases and experience. The results support further prospective evaluation of PE-MHL in clinical feline patients.

## Background

Feline intervertebral disc disease (IVDD) has been historically considered an uncommon disease with a low incidence (0.12–0.26%) [1–5]. However, the reported incidence has been increasing in recent years (0.44%), possibly due to more widely available advanced diagnostic imaging and higher proportion of insured pets able to access these modalities [6]. IVDD ranks second to aortic thromboembolism as a cause of acute pelvic limb paralysis in cats [7]. Among the different types of IVDD, intervertebral disc extrusion (IVDE) is most frequently observed, with the thoracolumbar and especially the lumbar spine, being the most affected region [1, 3–8]. The typical clinical signs of acute disc herniation in cats include spinal pain, paralysis, and loss of voluntary urination [1, 4, 6, 8]. Intervertebral disc extrusions in cats may be treated conservatively or surgically [6]. Surgical treatment options for cats include hemilaminectomy (HL) [1, 3, 4, 8, 9] laminectomy [10] and foraminotomy [11]. Hemilaminectomy, the most used surgical treatment technique in dogs as well as cats, provides good access to the spinal canal and spinal cord [1, 3, 4, 8] [9, 12]. Mini-hemilaminectomy (MHL), an alternative technique, has so far only been reported in dogs [13–15]. Compared to HL, the method of MHL has the advantage of preserving the articular process and is therefore less likely to result in mechanical instability of the spine [16–19]. Furthermore, the technique may reduce morbidity due to its lesser invasiveness [20]. On the other hand, due to the smaller bone window created MHL may be selected for ventrolateral disc extrusions or lesions involving the intervertebral foramen and may limit the access to the dorsal and dorsolateral vertebral canal [15]. Mini-hemilaminectomy can be performed via an open surgical approach or minimal invasively [21, 22]. Generally, for minimally invasive spine surgery (MISS) of the thoracolumbar spine in companion animals, percutaneous endoscopic [21, 23–25] or techniques using tubular systems have been reported [22, 26–31]. Overall, the primary goal of MISS is to minimize iatrogenic trauma, while achieving the equivalent therapeutic outcome as more invasive techniques [32]. Reduced tissue trauma may result in preserved spinal stability, decreased morbidity, a shorter recovery period and reduced hospital stay [32]. Although, these techniques aim to shorten surgical time, thereby lowering the risk of infection and anesthesia-related complications, an initial steep learning curve can be a drawback for surgeons [32]. While MISS is common in human medicine, veterinary MISS research is largely limited to canine patients, apart from a single case report in a cat [21–31, 33]. PE-MHL has so far only been reported in a single cadaveric canine study [21].

The aim of this cadaveric study is to evaluate the feasibility and prospective advantages of percutaneous endoscopic mini-hemilaminectomy (PE-MHL) and to compare it to standard open mini-hemilaminectomy (SO-MHL) in cats.

Our main hypothesis is that PE-MHL would be technically feasible in the feline lumbar spine. Additionally, in a cadaveric setting, the authors expected PE-MHL to yield comparable procedural metrics (surgical time, bone window size, complications) to SO-MHL, while demonstrating the technical features typical of minimally invasive approaches (wound size).

## Methods

### Preliminary test

**Fig. 1:**
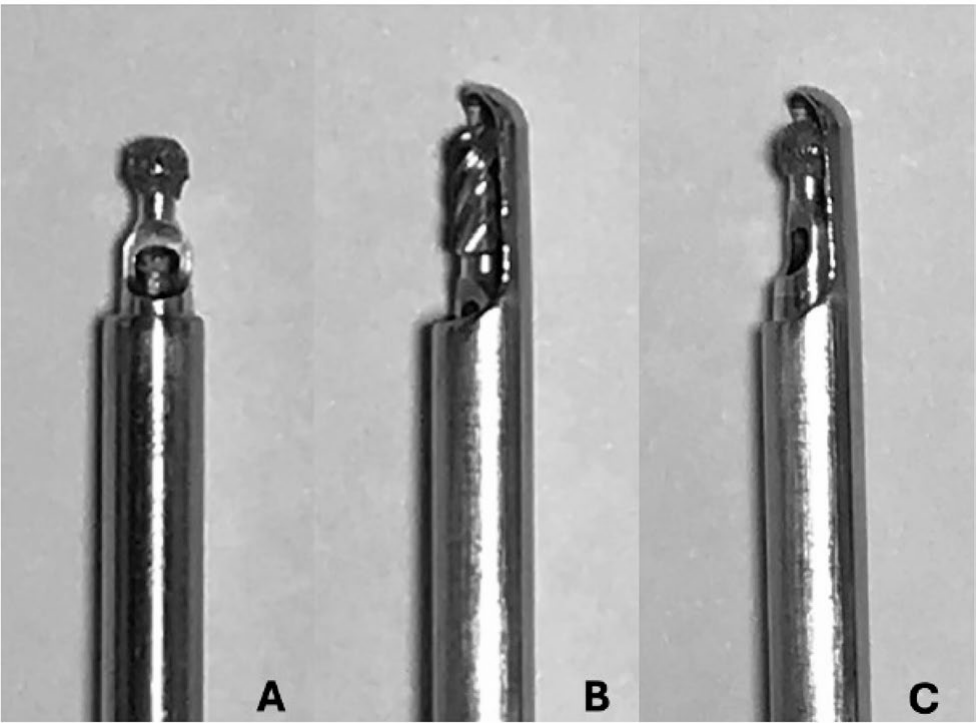
Burrs of the SpineTIP system (Karl Storz, Germany) and adjustment made for percutaneous endoscopic mini-hemilaminectomy in cats. Diamond burr (**A**), cutting burr combined with a protecting sheath (**B**), Diamond drill bit combined with the protecting sheath of the suction burr **C**

Preliminary investigations were conducted using three cadavers to allow familiarization with the equipment and the procedure of PE-MHL. The three cadavers included one male and two female cats, with a median body weight of 4.2 ± 0.75 kg (range 3.5–5.0 kg). According to published reports, the lumbar spine in cats is disproportionately affected by IVDE [5, 6, 8], with a peak incidence at the L4-L5 intervertebral disc space [34]. Furthermore, morphometric data indicate that the mid-lumbar region, including L4–L5, has consistent anatomical landmarks, making it favourable for reproducible cadaveric procedures [35]. Therefore, the authors elected to focus on the lumbar region, specifically the L4-L5 segment, for this study. Localization of the intervertebral disc space, approach and initial burring of a foraminotomy were tested at the L4-L5 region. The percutaneous endoscopic mini-hemilaminectomy was performed using the Spine TIP system (interlaminar approach) by Karl Storz (Germany). This system provided suction burrs with a cutting drill bit (2.2 mm width and 4.5 mm length, 3.5 mm outer diameter of the shaft). These were combined with a protecting sheath on one side of and at the tip of the drill (Fig. 1B).

Alternatively, diamond burrs with distally unsheathed round drill bits (2.2 mm diameter, 3.5 mm outer diameter of the shaft) were available (Fig. 1A). The latter were recommended by the manufacturer for foraminotomies. However, to avoid damage of the spinal cord and nerve root, the sheathed drill was prioritized. It was observed that using the cutting drill in a side-to-side manner for lamina removal would necessitate advancing the drill too far into the vertebral canal, which risked compressing the spinal cord. In addition, the entire length of the drill could not be visualized, potentially injuring critical structures. Therefore, the diamond burr was combined with the sheath of the suction burr (Fig. 1C). With this adjustment the lamina could be removed under complete visualization protection of the spinal cord.

### Set up and planning

In accordance with the findings of the preliminary assessment of PE-MHL, cadavers of additional 15 domestic short-haired (DSH) cats were collected for this study. The animals were client-owned cats that died or were euthanized for reasons unrelated to the study and were determined to be free of a neurological condition at the time of death. Inclusion criteria were breed (DSH), skeletal maturity and the absence of spinal disease. Orthogonal radiographs (Siemens Luminos dRF Max) of the thoracolumbar vertebral columns were performed to exclude obvious bony pathologies such as vertebral fractures, spondylosis deformans or degenerative joint disease. All included cadavers were frozen at −20 °C. Prior to the study, the cadavers were thawed to room temperature for 24 h.

To evaluate procedure times as a potential factor of procedural improvement while minimizing side-related variability, PE-MHL was performed on the left and SO-MHL on the right side of the L4-L5 intervertebral disc space. The cats were clipped, placed in sternal recumbency, fixed to the table and surgically draped to mimic realistic conditions. All procedures were performed by one board-certified surgeon (MK) over a period of 1 week.

In order to achieve comparable bone windows, the lengths of the L4 and L5 laminae from the mid of the intervertebral foramen were measured on calibrated lateral radiographs of the spine. The mean lengths of L4 and L5 were 18.0 mm and 18.5 mm respectively. The removal of approximately one-third of the respective lamina was planned. According to the measurements of the radiographs this was estimated to be approximately 6 mm of each lamina in a cranial (L4) and caudal direction (L5). The width of the dissector (2.8 mm) served as a reference during surgery.

### Surgical procedure: percutaneous endoscopic mini-hemilaminectomy

**Fig. 2:**
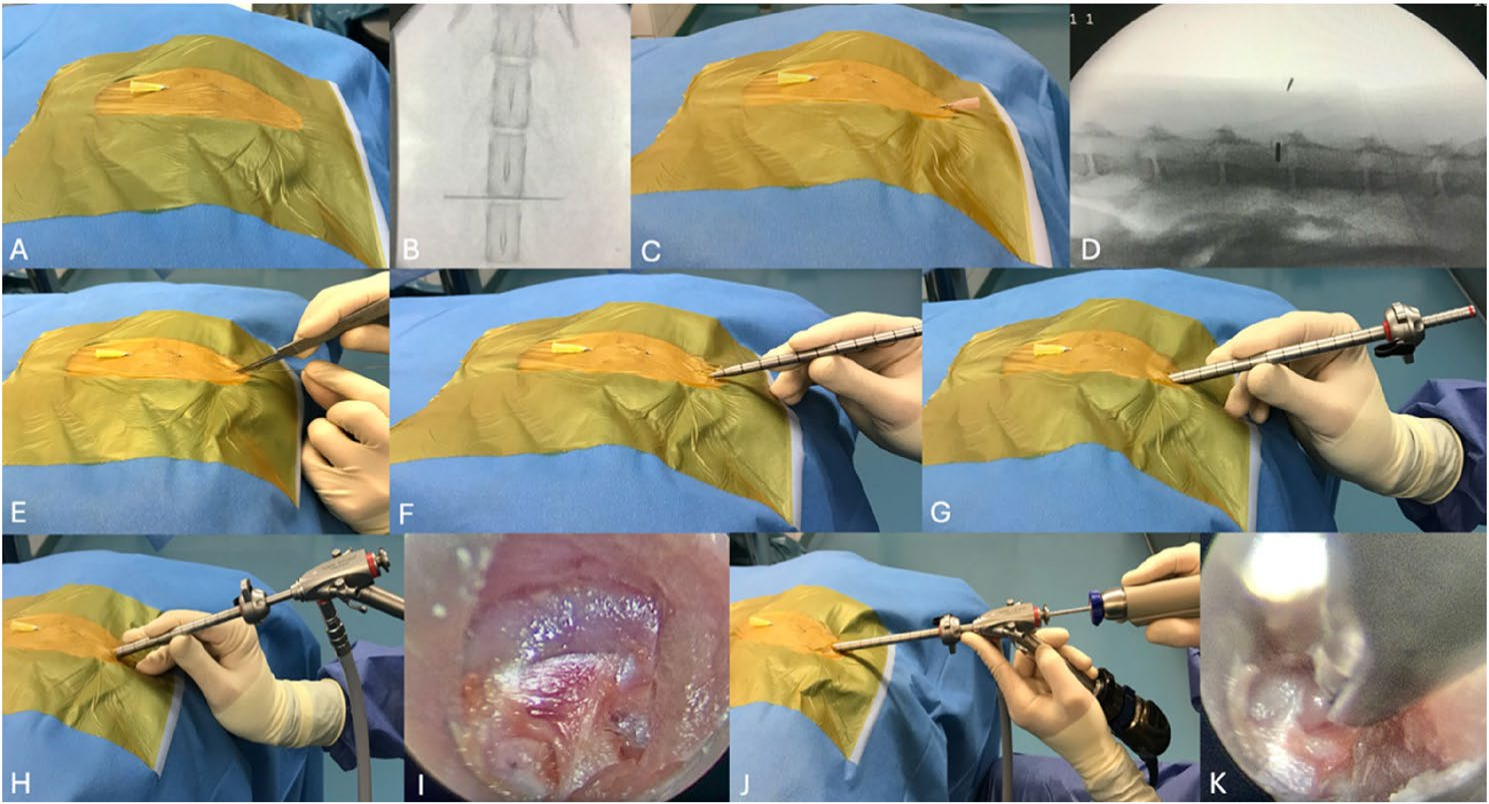
Step-by-step illustration of the surgical procedure (PE-MHL). **A** dorsal localization and placement of a 20G needle, (**B**) dorsal fluoroscopic confirmation, (**C**) lateral localization and placement of an 18G needle, (**D**) lateral fluoroscopic confirmation, (**E**) guide-wire insertion and skin incision, (**F**) advancement of the dilation sleeve, (**G**) positioning of the operating sheath, (H) removing of the dilation sleeve and insertion of the Hopkins telescope, (**I**) endoscopic view of the intervertebral foramen, (**J**) insertion of the Spine handpiece with diamond burr and protective sheath, (**K**) initial burring of the lamina with partial exposure of the vertebral canal

For the dorsal identification of the surgical site in both procedures, spinous processes were counted starting caudally from L7. The intervertebral disc space of the L4-L5 segment was marked by inserting a 20G needle between the spinous processes (Fig. 2A) and confirmed fluoroscopically with dorsoventral images via a mobile C-arm (Cios Spin, Siemens Healthineers) starting at the level of the pelvis, continuing cranially whilst counting the vertebrae (Fig. 2B). Consecutively, for lateral identification of surgical site in PE-MHL, the facet on the left side was palpated and a 18G needle was inserted on the cranial lamina centered to the intervertebral foramen (90° angle) (Fig. 2C). The position was confirmed fluoroscopically with lateral images (Fig. 2D).

**Fig. 3:**
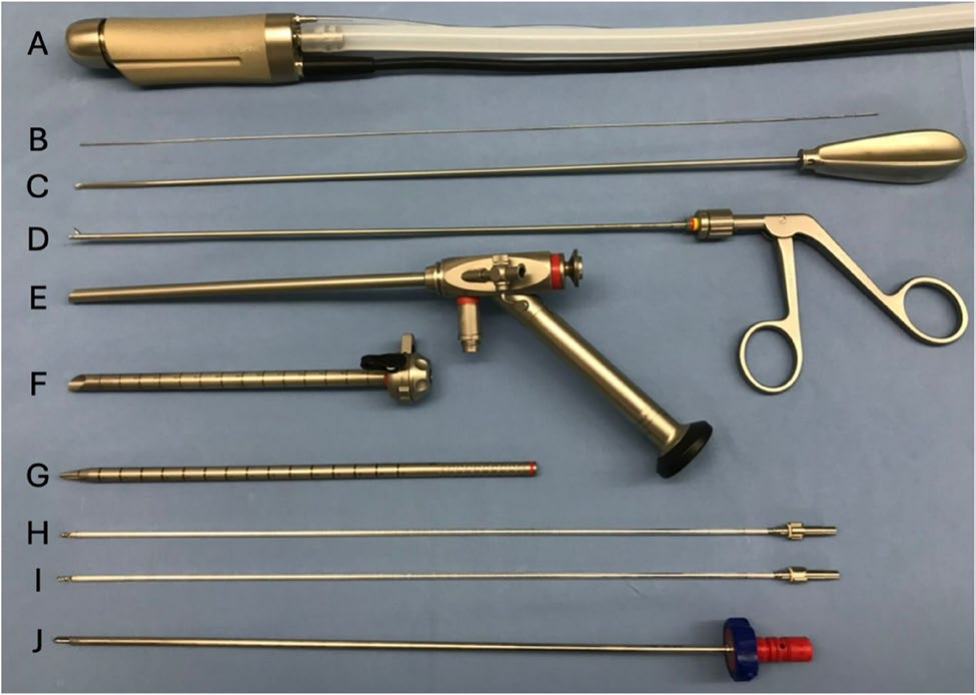
Endoscopic set-up. **A** Spine handspiece (DrillCut-X II SPINE), (**B**) guide wire, (**C**) dissector, (**D**) grasping forceps, (**E**) Hopkins telescope, (**F**) distal oblique operating sheath, (**G**) dilatation sleeve, (**H**) diamond burr, (**I**) cutting burr, (**J**) protecting sheath

PE-MHL was performed using the previously tested SpineTIP system. A guide wire (0.7 mm diameter, 410 mm length) (Fig. 3B), a dilation sleeve (6.5 mm outer diameter, 1.4 mm inner diameter, 22 mm length, 2-channel) (Fig. 3G) and a distal oblique operating sheath (7.9 mm outer diameter, 7.2 mm inner diameter, with a fixation mechanism for the endoscope) (Fig. 3F) were used to approach the intervertebral foramen. A Hopkins telescope (6.6 mm outer diameter, 180 mm length, 25° vision angle and a working channel with a diameter of 3.6 mm) (Fig. 3E) with an inlet for light was introduced and fixed to the operating sheath. A Spine handpiece (DrillCut-X II SPINE) (Fig. 3A) with inlets for rinsing (silicone tubing set for irrigation) and suction was connected to a Unidrive S III Neuro SCB. Optionally, a diamond burr (3.5 mm outer diameter of the shaft) (Fig. 3H) or a suction burr (3,5 mm outer diameter of the shaft) (Fig. 3I) were available to be used with the Spine handpiece. Based on the preliminary testing, the burrs originally developed for human surgery were modified to be used in this study. A dissector (2.8 mm diameter, 360 mm length) (Fig. 3C) and a grasping forceps (2.7 mm diameter, 300 mm length) (Fig. 3D) were also used.

**Fig. 4:**
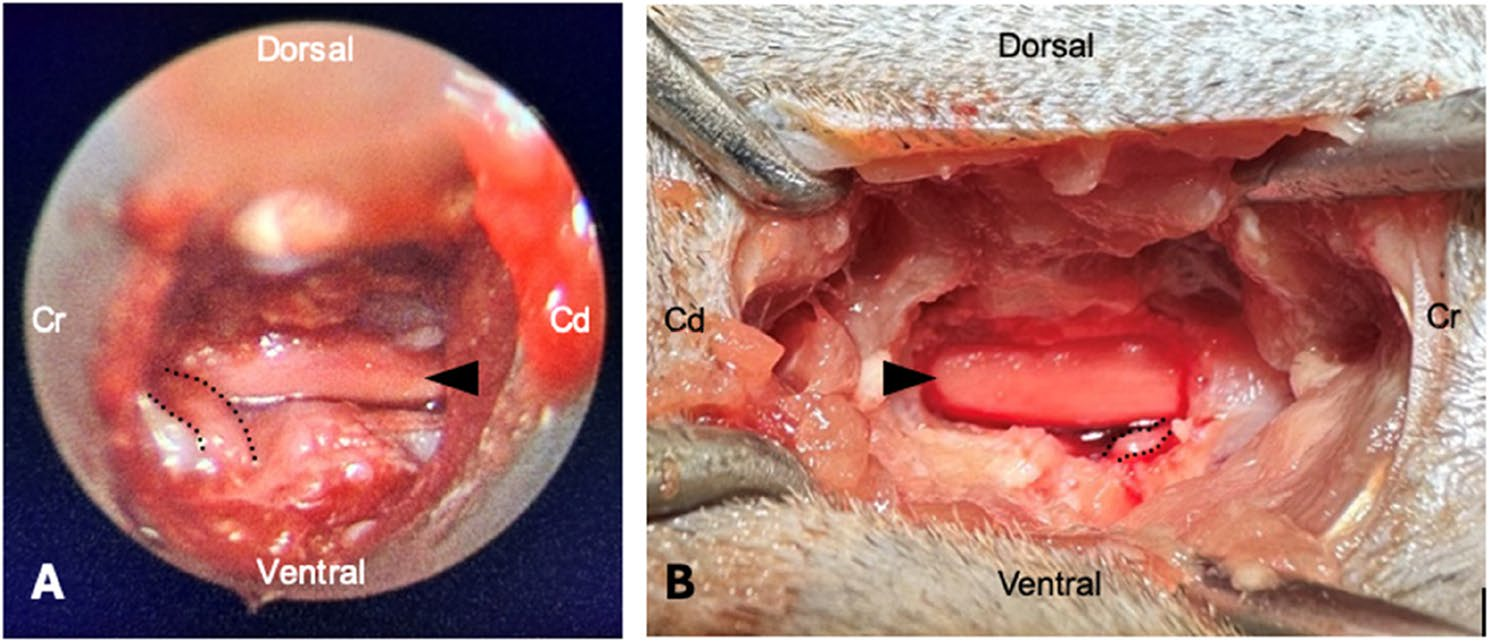
Exemplary images of left-sided PE-MHL (**A**) and right-sided SO-MHL (**B**) of the lumbar spine at the level of L4-L5 in a cadaveric cat. The spinal cord (black arrowhead) and the spinal nerve (black dotted line) are clearly visualized and the ventral floor of the spinal canal was accessible

The guide wire was inserted into the 18G needle, which was subsequently removed. Maintaining the contact of the guide wire with the lamina, the skin and fascia were incised via a stab incision. The dilation sleeve was introduced over the guide wire until bony resistance was detected. The guide wire was removed and the operating sheath was placed over the dilation sleeve, which was subsequently removed. The telescope was inserted and fixed to the operating sheath. The accessory process served for orientation. The beveled operating sheath was placed cranially to the cranial vertebra and caudally to the caudal vertebra for adequate visualization while clearing away the epaxial musculature from the lamina using a dissector and grasping forceps, until the intervertebral foramen as well as the cranial and caudal lamina could be identified. The speed rate of the burr was set according to the recommendation of the manufacturer (2500 rpm). A flow rate of 3 (scale 1–9) was used for irrigation and an appropriately adjusted suction intensity allowed for satisfactory burring and visualization. The sheathed diamond burr, connected to the handpiece, was passed through the working channel of the telescope and advanced to the intervertebral foramen. The cranial and caudal lamina were removed under continuous irrigation with saline solution, as described above (Fig. 4A). Layered wound closure was performed using a simple interrupted pattern for the fascia (Monoplus 4/0, Braun SE, Germany), the subcutaneous tissue (Monosyn 3/0) and the skin (Optilene 4/0).

### Surgical procedure: standard open mini-hemilaminectomy

For the identification of the surgical site the 20G needle between the spinous processes of L4-L5 was left in place and the time measurements were not taken again as they were expected to be the same as for PE-MHL. Consecutively, the facet on the right side was palpated and a modified lateral approach as described by Tanaka was performed [36]. Gelpi retractors were used for tissue elevation. A mini-hemilaminectomy was performed according to the reported landmarks [13–15]. The accessory process was removed and its dorsal margin marked the dorsal extent of the mini-hemilaminectomy. Ventral to this, the pedicle was removed in a cranial and caudal direction from the intervertebral foramen as previously defined. Ventrally the mini-hemilaminectomy extended to the ventral aspect of the intervertebral foramen. A high-speed power drill (Electric Pen Drive, DePuy Synthes, Oberdorf, Switzerland) with cutting drill bits (diameter of 3 and 4 millimeters) under continuous irrigation was used. Burring was continued until inner cortex was exposed. The inner cortex was removed using a Kerrison rongeur (Fig. 4B). Layered wound closure was performed using a simple continuous pattern for the fascia (Monoplus 4/0 B. Braun SE, Germany) and subcutaneous tissue (Monosyn 3/0 B. Braun SE, Germany) and a simple interrupted pattern for the skin (Optilene 4/0 B. Braun SE, Germany).

### Assessment

The surgeries were considered successful if the standard and MISS mini-hemilaminectomies extended over approximately one-third of the respective laminae, the spinal cord as well as the spinal nerve were visible, and the ventral floor of the vertebral canal could be probed along the entire length of the bone window. Complications occurring during the procedures and any required adjustments were documented.

The surgical techniques were compared using several criteria including procedure time, laminectomy size and visual laminectomy site assessment.

The total procedure time was measured and further divided in four sections: fluoroscopy (identification of the surgical site), surgical approach, spinal cord exposure (creation of the bone window), and closure. For PE-MHL the duration of fluoroscopy included the time for both the dorsoventral and lateral identification of the surgical site.

**Fig. 5:**
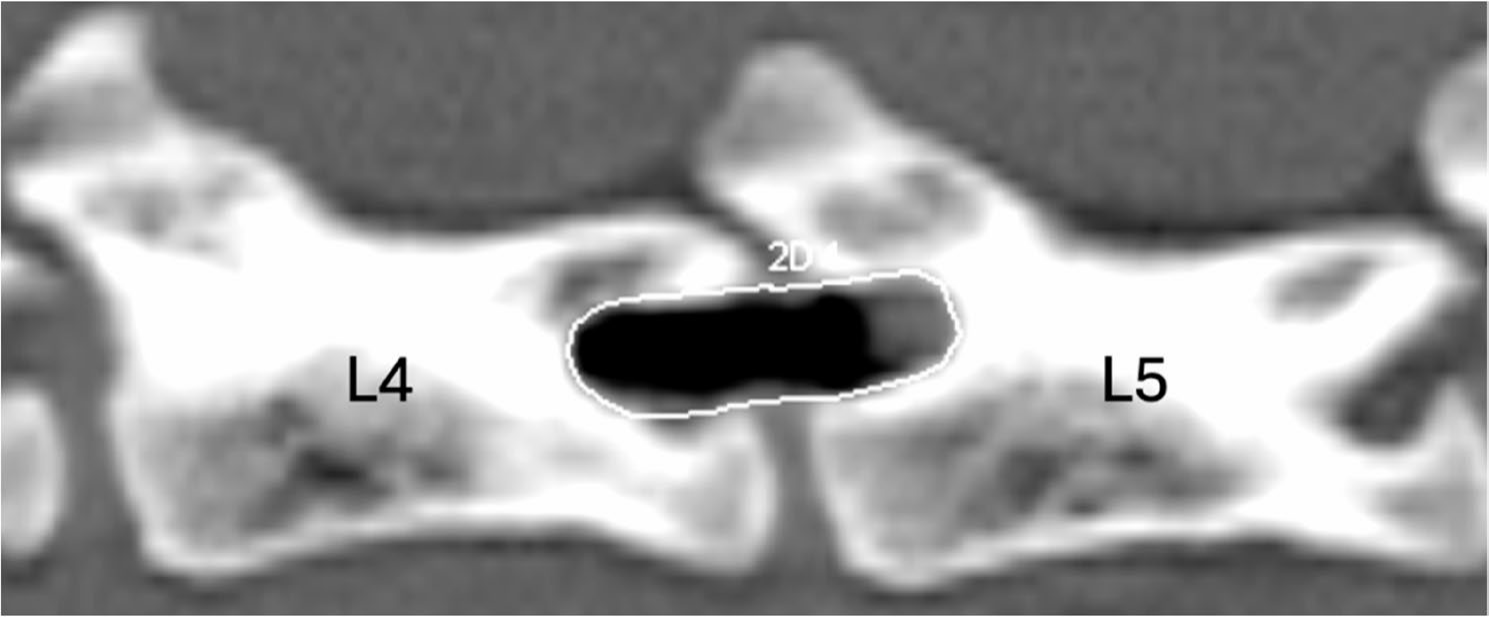
CT image of cat 13 at the L4-L5 level after left-sided PE-MHL. The outlined area represents the measured bone window with a calculated surface of 50 mm²

After surgery, the incision size (mm) following the removal of retractors and endoscope were measured using a ruler. The time measurement was paused for this assessment. Postoperatively, a computed tomography (CT) scan of the spinal segment (L3-6) was performed with a 64-lines multi-slice device (Siemens SOMATOM Definition AS 64), 300 mAs, 120 kV, and 0.6 mm slice thickness. The area of the created bone window (mm²) was determined based on its length and height, as well as circular measurements. Due to the irregular shape of the bone windows, the authors believed the circular measurements could provide more meaningful data and were therefore used for comparison of the surgical methods (Fig. 5).

For the circular measurements, points were placed along the resected bony margin of the window with varying distances as required for precision and automatically connected by lines. This procedure allowed precise alignment with the bone contour. The enclosed area was automatically calculated by the software as the circular area. These measurements were performed in the sagittal plane using the bone window. The software version used for the calculations was Somaris/7 syngo CT VA48A.

Finally, the surgical site was reopened (SO-MHL) or extended (PE-MHL), and assessed for iatrogenic trauma affecting the spinal cord or spinal nerves by direct visual inspection, using a probe when necessary. The evaluation focused on macroscopic laceration, disruptions or visible indentations.

### Statistical analysis

All statistical analyses were performed using R (version 4.4.1) software. For the evaluation of the parameter (times of total procedure, identification of surgical site, approach, exposure, closure, size of approach and bone window) mixed effect models (MEM) with interaction between surgical method and timepoints were performed. The assumptions underlying the MEMs were checked as followed. Normality of residuals was tested using the Shapiro-Wilk test. Homoscedasticity was assessed via the Breusch-Pragan test. Multicollinearity among predictors was examined using the Variance Inflation Factor (VIF). Potential influential data points were identified using Cook’s Distance. Robust MEM was applied, when assumptions were not satisfied. The results were considered statistically significant if the p value was equal to or less than 5% (*p* ≤ 0.05). All numerical values are expressed as mean ± standard error of the mean (SEM). Data are presented this way to indicate the precision of the estimated mean and facilitate comparison between groups. While SD reflects the variability within a dataset, SEM provides an estimate of how far the sample mean is likely to be from the true population mean [37]. Given the paired cadaveric study design, SEM was chosen to better reflect the accuracy of the mean differences reported.

The sample size of 15 specimens was determined in accordance with recommendations for pilot studies, which suggest that approximately 10–15 specimens per group are sufficient [38, 39]. Therefore, a power analysis was not performed.

## Results

A total of 15 cadavers of DSH cats were included in this study. Nine cats were male and six were female, with a median body weight of 3.9 ± 0.18 kg (range 3.0–5.5 kg). Fifteen left-sided PE-MHLs and 15 right-sided SO-MHLs were performed at the level of L4-L5. The mean total procedure time for the PE-MHLs was 65.40 ± 5.51 min, while the SO-MHLs took on average 30.30 ± 0.20 min (Table 1).

**Table 1:** Mean ± SEM values for operative times and tissue dimensions in 15 feline cadavers undergoing percutaneous endoscopic mini-hemilaminectomy (PE-MHL) and standard open mini-hemilaminectomy (SO-MHL)

	Localization (min)	Approach (min)	Exposure (min)	Closure (min)	Total procedure (min)
PE-MHL	2.28 ± 0.18	5.12 ± 0.31	55.49 ± 3.22	1.45 ± 0.06	65.40 ± 3.51
SO-MHL	1.04 ± 0.05	3.14 ± 0.11	21.08 ± 0.18	5.09 ± 0.08	30.30 ± 0.20
p value	< 0.001	0.002	< 0.001	< 0.001	< 0.001

Statistically significant at p ≤ 0.05

Values reported are mean ± SEM

The MEM revealed an estimated difference of 35.1 min between groups, which was significant (95% CI [43,4, 26.9], *p* < 0.001). The mean time required for identifying the surgical site was 2.28 ± 0.18 min for PE-MHL and 1.04 ± 0.05 min for SO-MHL (estimated difference 1.45 min, 95% CI [1.9, 1.0], *p* < 0.001)​. The duration of the surgical approach was 5.12 ± 0.31 min for PE-MHL and 3.14 ± 0.11 min for SO-MHL (estimated difference 2.05 min, 95% CI [3.23, 0.87], *p* = 0.002)​. Mean spinal cord exposure time was 55.49 ± 3.22 min for PE-MHL and 21.08 ± 0.18 min for SO-MHL (estimated difference 34.8 min, 95% CI [42.1, 27.6], *p* < 0.001)​. Mean closure time was 1.45 ± 0.06 min for PE-MHL and 5.09 ± 0.08 min for SO-MHL (estimated difference 3.39 min, 95% CI [3.01, 3.77], *p* < 0.001)​.

The mean length and width of the approach for the PE-MHL were 8.47 ± 0.20 mm and 9.53 ± 0.30 mm, respectively. For SO-MHL, the values were 22.09 ± 0.50 mm and 9.80 ± 0.30 mm. The estimated difference was 14.4 mm (95% CI [13.3, 15.6], *p* < 0.001) for approach length, and 0.27 mm (95% CI [0.65, 1.18], *p* = 0.54)​ for approach width. The mean closure length was 11.40 ± 0.50 mm for PE-MHL and 23.13 ± 0.70 mm for SO-MHL (estimated difference 11.7 mm, 95% CI [9.84, 13.6], *p* < 0.001)​.

### Intraoperative observations

PE-MHL was successfully performed in all cats. The spinal cord, as well as the spinal nerve were visible. The ventral floor of the spinal canal could be probed along the entire length of the bone window.

The first four procedures were considered the most challenging to perform. Particularly, endoscopic visualization and finding a balance between irrigation flow rate and suction intensity were the most difficult aspects of the surgery. Furthermore, burring in the cranial aspect of the vertebral foramen of the fourth lumbar vertebra and toward the center of the lamina of the fifth lumbar vertebra required additional time, which was not quantitatively recorded but only subjectively observed by the surgeon.

### Postoperative CT scan findings

The mean circular size of the bone window created by PE-MHL was 51.33 ± 1.65 mm², whereas that of SO-MHL was 64.67 ± 2.19 mm² (estimated difference 13.3 mm², 95% CI [8.19, 18.5], *p* = 0.001)​.

### Complications

During the burring process in PE-MHL, damage to the spinal nerve root occurred in one case (cat number 2). No further injuries to the spinal nerve or spinal cord were observed in either surgical method.

### Procedure times across cases

**Fig. 6:**
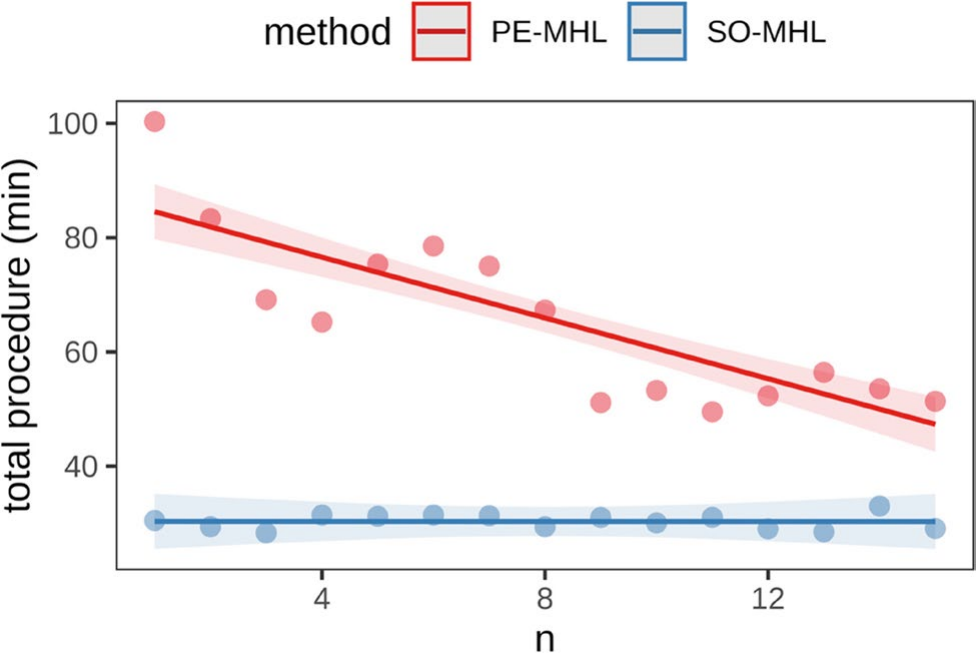
Illustration of the total procedure times (minutes) in relation to the number of the two surgical methods (PE-MHL, SO-MHL)

The PE-MHL exhibited a significantly longer procedure time compared to the SO-MHL (65.4 ± 5.51 min vs. 30.3 ± 0.20 min, *p* < 0.001). A significant reduction (*p* < 0.001) in procedure time was documented with increasing familiarity of the procedure (Fig. 6).

A significant reduction of surgical duration is present after 15 procedures of PE-MHL.

**Fig. 7:**
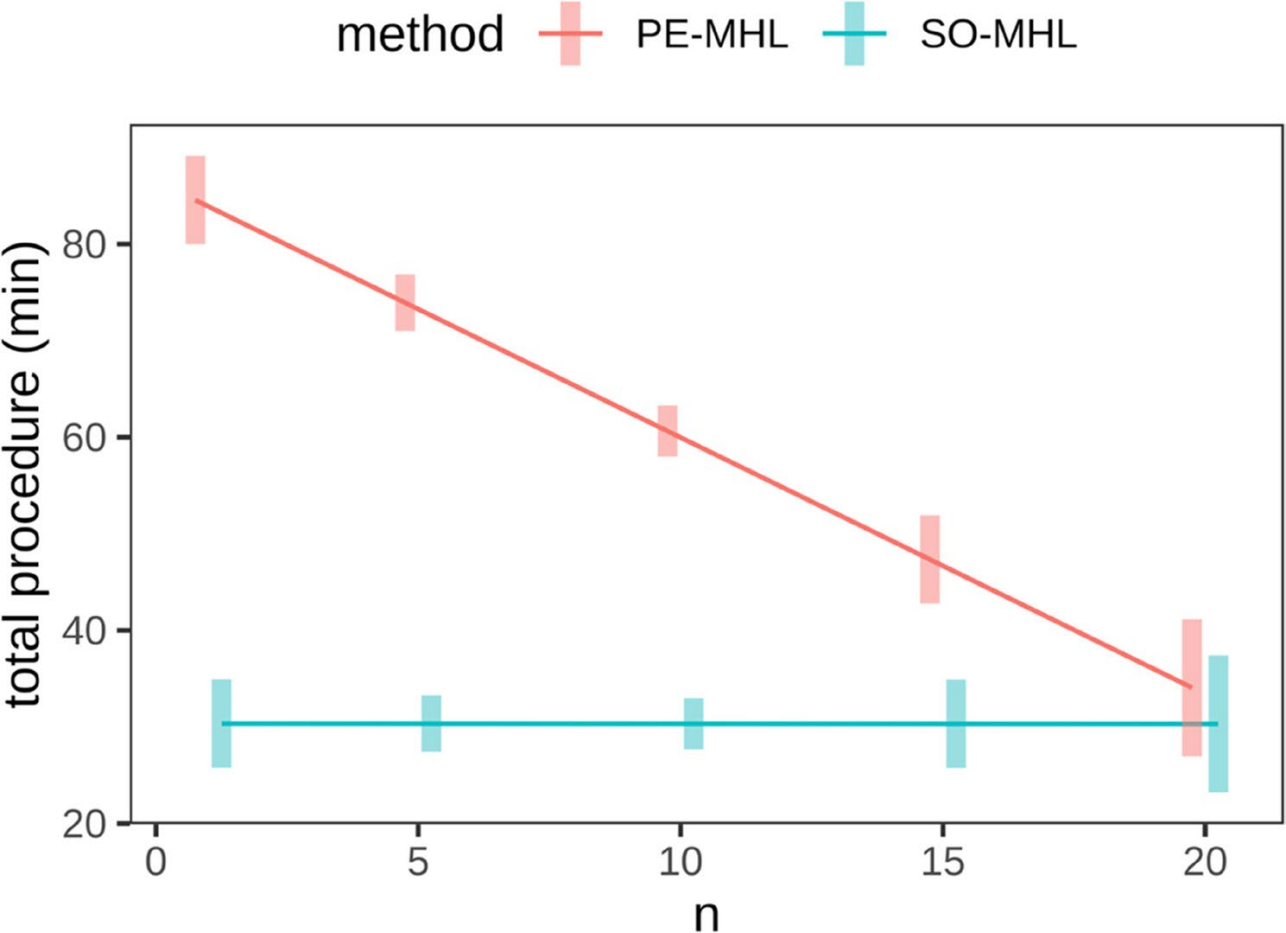
Illustration of procedure times of PE-MHL and SO-MHL across cases (n), including model-based estimates up to case number 20

Procedure time for PE-MHL was 84.5 min for the first case compared to 30.3 min for SO-MHL, and progressively decreased to 47.3 min at case number 15 (Fig. 7; Table 2). When procedure time was plotted against case number, a downward trend was observed. Model-based estimates indicated a mean procedure time of 34.1 min for PE-MHL after 20 cases, at which point no statistically significant difference to SO-MHL was detected (*p* = 0.464) (Fig. 7; Table 2).

**Table 2:** Procedure times and measurement data for PE-MHL and SO-MHL in 15 feline cadavers, including model-based estimates up to case number 20

*n*	Method	Mean time (min)	Difference (min)	*p* value
1	SO-MHL	30.3	−54.20	< 0.001
	PE-MHL	84.5		
5	SO-MHL	30.3	−43.58	< 0.001
	PE-MHL	73.9		
10	SO-MHL	30.3	−30.30	< 0.001
	PE-MHL	60.6		
15	SO-MHL	30.3	−17.02	< 0.001
	PE-MHL	47.3		
20	SO-MHL	30.3	−3.74	0.464
	PE-MHL	34.1		

Statistically significant at p ≤ 0.05

Observed procedure times are presented for PE-MHL and SO-MHL at cases 1, 5, 10, and 15. Model-based estimates are included for case number 20 to illustrate the trend in procedure time across cases

## Discussion

This study presents the first successful application of a full-endoscopic mini-hemilaminectomy in the vertebral column of feline cadavers providing a foundation for further clinical evaluation.

The results of this study demonstrate that PE-MHL is achievable in a feline cadaveric model and is expected to achieve comparable surgical times following 20 cases. The procedure offers potential advantages of MISS including decreased wound size and associated wound closure times as well as incision lengths. However, our initial assumption further was that bone window sizes would be comparable between techniques, but PE-MHL resulted in smaller bone windows. Therefore, these findings allowed only in part the acceptance of our hypothesis.

In the present study, a surgical approach of less than 1 cm was achieved. Moon et al. (2017) had already demonstrated the technical feasibility of a percutaneous endoscopic mini-hemilaminectomy using a uniportal approach and a skin incision of less than 1 cm in a cadaveric dog model [21]. Our findings confirm that in cats such a minimally invasive approach is also achievable. According to a canine study from Hettlich et al. (2017), the reduction of incision size is a key advantage of minimally invasive techniques as it is associated with a reduction in tissue trauma, fibrosis and decreased postoperative pain [32]. In humans, the use of MISS has been associated with shorter hospitalization times. It seems reasonable to assume that similar benefits could also be achieved in canine and feline patients. For example, a median hospitalization time of 4 days (range 3–7 days) has been reported in cats following open spinal decompression [8]. We therefore anticipate that the introduction of MISS techniques may help reduce hospitalization duration in feline patients. Further clinical studies in feline patients are needed to confirm these experimental findings and to evaluate clinical applicability and outcomes. Additionally, a smaller approach may promote earlier postoperative recovery.

Our results show that PE-MHL resulted in significantly smaller bone windows than SO-MHL (51.33 ± 1.65 mm² vs. 64.67 ± 2.19 mm²; *p* < 0.001). Despite this reduction, complete intraoperative visualization of the spinal cord and nerve roots through the bone window was possible. The ventral floor of the spinal canal could be fully explored along the length of the MHL. Moon et al. (2017) reported a mean bone window size of 63.37 ± 16.00 mm² at the comparable lumbar segment L2–3 and evaluated the efficacy of decompression with simulated intervertebral disc herniation in small dogs [21]. Their findings confirm that adequate visualization and decompression of the spinal canal, including the nerve roots, can be achieved through windows of this size. Importantly, the study also demonstrated that removal of simulated extruded disc material could be accomplished effectively through the endoscopic approach [21]. These results support the efficacy of PE-MHL for disc extrusion in dogs [21]. They further strengthen the assumption that the bone windows created in cats may likewise be sufficient for decompression when the disc material is centered over the intervertebral foramen. Although this still requires confirmation in clinical feline cases.

One spinal nerve injury occurred, which happened early in the study (cat number 2). Through adjustment of irrigation flow and suction power, excellent intraoperative visualization was achieved and further injuries could be avoided. In the cadaveric study by Moon et al. (2017) in 7 dogs with 14 surgeries two nerve root injuries occurred during the procedures in the thoracic spine [21]. This complication rate aligns closely with the results obtained in our study.

However, Hwang et al. (2016) described complications including possible MRI-detectable spinal cord changes and accessory process fractures in live dogs undergoing percutaneous endoscopic pediculectomy [23]. While such complications were not observed on our postoperative CT scans, specifically spinal cord pathology could have gone undetected.

In all PE-MHL procedures a diamond burr with a protective sheath was used. This enabled precise and controlled removal of the lamina under direct visualization, especially in areas in close proximity to the spinal cord. While laminar thickening toward the base of the facet and center of the vertebrae led to longer burring times, cutting burrs were deliberately avoided due to their higher risk of causing neural tissue damage. Although cutting burrs allow for faster removal of coarse bone, the diamond burr was chosen based on manufacturer recommendations and safety considerations. Further studies are warranted to evaluate whether the use of cutting burrs may also be feasible and potentially reduce surgical time without compromising vital structures.

In this cadaveric study, procedure time for PE-MHL decreased from 84.5 min at the first case to 47.3 min at case number 15, indicating that operative time served as a factor reflecting increasing familiarity with the technique. A full assessment of a learning curve associated with this procedure was beyond the scope of our study. To properly characterize a true learning curve, individual and group performances would need to be assessed, including aspects such as initial and final skill level, learning rate, error rate, and typical pitfalls. Nevertheless, when plotting procedure time against case number, a clear downward trend was demonstrated, suggesting that operative time may improve with repeated practice.

To ensure consistency in time measurements, PE-MHL was always performed on the left side and SO-MHL on the right side of the L4–L5 intervertebral space. This approach was chosen to ensure that each procedure was carried out with the same hand and side so that procedure times could be assessed more effectively without the added confounding factor of alternating sides. However, for a proper evaluation of surgical performance over time, potential side-related influences would need to be assessed separately.

This study has several limitations. It was performed exclusively on cadavers, restricting the extrapolation of results to clinical patients. Key aspects such as intraoperative bleeding, pain management, and postoperative healing could not be assessed. Furthermore, the current model could not simulate extruded disc material, thereby limiting evaluation of PE-MHL for such settings. The exclusive use of the L4–L5 intervertebral space further limits extrapolation. Vertebral canal dimensions vary substantially along the feline thoracolumbar spine. Richter et al. (2024) reported narrowing at L5–S1 and in the mid-thoracic spine, with the narrowest disc space often located at T10–T11 [35]. Such anatomical constraints may increase the technical difficulty of PE-MHL at these sites.

Future studies could evaluate the feasibility of PE-MHL at different, commonly affected, locations. A methodological limitation lies in the use of different burr types in the two groups: diamond burrs were used for PE-MHL and cutting burrs for SO-MHL. While this decision was based on manufacturer recommendations and the design of the cutting burrs, it may have influenced surgical time and precision, complicating direct comparisons.

Another limitation is that procedure time was the only parameter analyzed as an indicator of procedural improvement. While procedure times decreased across cases, this single outcome does not capture other important aspects such as error rates, intraoperative difficulties, or inter-surgeon variability. Furthermore, PE-MHL was always performed on the left and SO-MHL on the right side of the L4–L5 intervertebral space. Although this design ensured consistency in measurements, it may have introduced side-related bias. Randomization of sides would more closely replicate clinical conditions and should be considered in future studies.

Neither histopathological evaluation nor post operative MRI scanning was conducted to assess potential injury to the spinal cord or nerve roots, limiting assessment of iatrogenic trauma. Additionally, all PE-MHL procedures were performed by a single experienced surgeon. Although ensuring consistency, it limits applying our findings to less experienced surgeons. A multicenter design with multiple surgeons would be desirable to allow for better assessment of reproducibility and learning curve.

## Conclusions

In conclusion, this study demonstrates that percutaneous endoscopic mini-hemilaminectomy may be technically feasible in cats and, with increasing experience, may match the standard open technique in terms of surgical duration, with the added benefits of MISS. The results support further investigation of PE-MHL as a prospective alternative to SO-MHL.

## Data Availability

The datasets analysed during the current study are available from the corresponding author on reasonable request.
